# Hepatic-Specific Decrease in the Expression of Selenoenzymes and Factors Essential for Selenium Processing After Endotoxemia

**DOI:** 10.3389/fimmu.2020.595282

**Published:** 2020-11-05

**Authors:** Laura G. Sherlock, Kara Sjostrom, Lei Sian, Cassidy Delaney, Trent E. Tipple, Nancy F. Krebs, Eva Nozik-Grayck, Clyde J. Wright

**Affiliations:** ^1^ Perinatal Research Center, Department of Pediatrics, University of Colorado Anschutz Medical Campus, Aurora, CO, United States; ^2^ Perinatal Nutrition Laboratory, Department of Pediatrics, University of Colorado Anschutz Medical Campus, Aurora, CO, United States; ^3^ Cardiovascular Pulmonary Research Laboratories, Departments of Pediatrics and Medicine, University of Colorado Anschutz Medical Campus, Aurora, CO, United States; ^4^ Department of Pediatrics, University of Oklahoma College of Medicine, Oklahoma City, OK, United States

**Keywords:** selenium, selenoenzymes, liver, endotoxemia, selenoprotein P, selenocysteine processing, nutritional immunology

## Abstract

**Background:**

Selenium (Se) levels decrease in the circulation during acute inflammatory states and sepsis, and are inversely associated with morbidity and mortality. A more specific understanding of where selenoproteins and Se processing are compromised during insult is needed. We investigated the acute signaling response in selenoenzymes and Se processing machinery in multiple organs after innate immune activation in response to systemic lipopolysaccharide (LPS).

**Methods:**

Wild type (WT) adult male C57/B6 mice were exposed to LPS (5 mg/kg, intraperitoneal). Blood, liver, lung, kidney and spleen were collected from control mice as well as 2, 4, 8, and 24 h after LPS. Plasma Se concentration was determined by ICP-MS. Liver, lung, kidney and spleen were evaluated for mRNA and protein content of selenoenzymes and proteins required to process Se.

**Results:**

After 8 h of endotoxemia, plasma levels of Se and the Se transporter protein, SELENOP were significantly decreased. Consistent with this timing, the transcription and protein content of several hepatic selenoenzymes, including SELENOP, glutathione peroxidase 1 and 4 were significantly decreased. Furthermore, hepatic transcription and protein content of factors required for the Se processing, including selenophosphate synthetase 2 (Sps2), phosphoseryl tRNA kinase (Pstk), selenocysteine synthase (SepsecS), and selenocysteine lyase (Scly) were significantly decreased. Significant LPS-induced downregulation of these key selenium processing enzymes was observed in isolated hepatocytes. In contrast to the acute and dynamic changes observed in the liver, selenoenzymes did not decrease in the lung, kidney or spleen.

**Conclusion:**

Hepatic selenoenzyme production and Se processing factors decreased after endotoxemia. This was temporally associated with decreased circulating Se. In contrast to these active changes in the regulation of Se processing in the liver, selenoenzymes did not decrease in the lung, kidney or spleen. These findings highlight the need to further study the impact of innate immune challenges on Se processing in the liver and the impact of targeted therapeutic Se replacement strategies during innate immune challenge.

## Introduction

Sepsis is a major cause of morbidity and mortality (1–3). Immune dysregulation during sepsis can contribute to severity of disease, end organ injury and mortality (4). Micronutrient status is an important and modifiable aspect of the innate immune response. Selenium (Se) is one such micronutrient that contributes to immune health, influencing redox state as well as the acute and chronic inflammatory responses (5, 6). Se exerts its biologic effect after incorporation into selenoenzymes that provide antioxidant defense during exposures associated with oxidative stress, such as sepsis (5, 7). Several of these selenoenzymes are implicated in intracellular signaling and immune cell function, and contribute to the inflammatory response after injury (8–10). Insufficient selenoprotein defense exacerbates the inflammatory response in endothelial cells and macrophages, and worsens the pathogenicity and mortality after viral and bacterial infections in rodent models (11–19).

Selenium homeostasis undergoes dynamic changes during innate immune challenges. Circulating levels of Se and the Se enriched transporter protein selenoprotein P (SELENOP) decrease in septic and critically ill adults, and are inversely correlated with the risk of multiorgan dysfunction, pneumonia, and death (20–24). In murine models, innate immune activation with endotoxemia decreases circulating Se and SELENOP levels, mimicking the clinical paradigms (25, 26). Collectively, these findings provided the rationale for a series of randomized clinical trials of Se replacement to improve outcomes in critically ill adults. Unfortunately, these trials were unsuccessful at improving mortality or changing the inflammatory response (27–30). The lack of clinical effect and limitations in these studies highlight gaps in knowledge related to the function and regulation of Se during innate immune challenge.

While it is established that sepsis and innate immune activation with endotoxemia decrease circulating Se, the impact of decreased circulating Se on Se availability in various organs is incompletely understood (25, 26). This is important as selenoenzyme status in organs including the lung, kidney and spleen modulates inflammation and oxidative stress after injury (31–33). Additionally, it is unclear if Se processing pathways remain during innate immune stimulation. To convert dietary or supplemental Se to its bioactive form, Se must be co-translationally incorporated into the 21^st^ amino acid, selenocysteine (Sec), a complex multistep process that requires multiple enzymatic conversions and proteins to load Se into its specific Sec tRNA (34). Prior work demonstrated endotoxemia decreases the hepatic transcription for multiple biofactors required for Sec synthesis (25). However, it has not been described if this corresponds with protein level changes that could potentially limit the utilization of Se or generation of selenoproteins after endotoxin exposure.

To improve our understanding of the mechanisms and kinetics of Se regulation after innate immune challenge, we interrogated organ-specific responses in selenoenzymes and factors required for Se processing after inflammatory insult. We used a murine model of innate immune challenge with endotoxemia and tested the acute signaling response of selenoenzymes and factors involved in Se processing in the liver, lung, kidney, and spleen. We found that in endotoxemic adult mice, selenoenzymes and factors required to incorporate Se into Sec decreased significantly in the liver. In contrast, we did not observe decreases in key selenoenzymes the lung, kidney and spleen after endotoxemia. These results highlight the need to further define the impact of an acquired decrease in selenoenzyme defense in the liver after innate immune activation.

## Materials and Methods

### Murine Model of Endotoxemia

Adult (8–12 weeks, male) C57Bl/6J mice from Jackson labs were exposed to lipopolysaccharide (LPS) (Sigma L2630, 5 mg/kg, intraperitoneal (IP). Unexposed controls were compared to animals exposed to LPS for 2, 4, 8 or 24 h. All procedures were approved by the IACUC at the University of Colorado (Aurora, CO). Care and handling of the animals was in accord with the National Institutes of Health guidelines for ethical animal treatment.

### Collection of Blood and Organs

Mice were euthanized with a fatal IP dose of pentobarbital sodium (200 mg/kg, IP). Blood was removed by right ventricular heart puncture through a closed chest, collected in heparinized tubes and placed on ice. These samples were then spun at 3,000 rpm x 10 min at 4°C. Plasma was aliquoted and stored at −80°C. The pulmonary artery was perfused with 10 ml phosphate buffered saline (PBS) to flush the organs, and liver was visualized to assure blanching. Organs were then removed, snap frozen and stored at −80°C.

### Selenium Content by Inductively Coupled Plasma Mass Spectrometry (ICP-MS)

Plasma samples (50µl) were subjected to inductively coupled plasma mass spectrometry, measuring ^82^Se because of its low background noise. Plasma and Se standards were prepared in 2% nitric acid (HNO_3_) containing approximately 6 parts per billions rhodium (Aldrich Chemical Company, Inc., Milwaukee, WI) and 0.1% Triton X-100. A set of Se standards was inserted every 10 samples for external drift correction. Seronorm Trace Elements Serum L-1 (SERO, Billingstad, Norway) was used at the beginning and the end of each run to check the accuracy of measurements.

### ELISA

Plasma levels of glutathione peroxidase 3 (Gpx3) were measured by ELISA (LSBio, Seattle, WA) according to manufacturer’s instructions.

### Glutathione Peroxidase Activity

Plasma glutathione peroxidase was determined by measuring a glutathione reductase-coupled test as previously described (35). Plasma was collected as described above. Plasma was diluted to a 1:5 dilution with deionized water. Samples were incubated with reaction buffer for 10 min at 37°C, then hydrogen peroxide was added at a final concentration of 50 μM. The reaction was monitored for 2 min at 340 nm on a microplate reader. Glutathione peroxidase activity was then calculated using Lambert-Beer’s law, with 1 U of activity defined as the consumption of 1 μmol NADPH/min/ml.

### Immunoblot Analysis

Liver was lysed in TPER buffer with Halt protease and phosphatase inhibitors (ThermoFisher, Waltham, MA) (1:100). Lysates were spun at 14,000 g for 10 min, supernatant was removed, and protein content was determined by Pierce BSA assessment (ThermoFisher, Waltham, MA). 30–50ug of protein was electrophoresed on a 4–12% polyacrylamide gel (Invitrogen, Waltham, MA) and proteins were transferred to an Immobilon membrane (Millipore, Burlington, MA). Blots were then exposed to the following antibodies: Glutathione peroxidase 1 (1:1,000, R&D Systems, Minneanapolis, MN), Glutathione peroxidase 4 (1:500, Santa Cruz, Santa Cruz, CA), Pstk (1:500, LSBio, Seattle, WA), Selenocysteine Lyase (1:1,000, Santa Cruz, Santa Cruz, CA), SELENOP (1:1,000, generous gift from Dr. Yoshiro Saito and Dr. Hiraoki), SepsecS (1:500, LSBio, Seattle, WA), Selenophosphate Synthetase 2 (1:1,000, Rockland Institute, Limerick, PA), Thioredoxin reductase 1 (1:2,000, TE Tipple lab, Oklahoma City, OK), Thioredoxin reductase 2 (1:1,000, Santa Cruz, Santa Cruz, CA). Secondary antibodies were in the appropriate host (1:5,000). Blots were imaged utilizing the LiCor Odyssey system. Densitometry analysis was performed using ImageStudio (LiCor, Lincoln, NE).

### Analysis of Relative mRNA Levels by RT-qPCR

Frozen tissue was placed in RLT buffer (Qiagen) and homogenized using the Bullet Blender (NextAdvance, Troy, NY). Hepatic, pulmonary, renal and splenic mRNA were isolated using RNeasy Mini Kit (Qiagen, Germantown, MD) according to the manufacturer’s instructions. RNA was assessed for quality and concentration by Nanodrop (ThermoFisher Scientific, Waltham, MA). cDNA was synthesized at 1μg/20μl using Verso cDNA synthesis kit (ThermoFisher Scientific, Waltham, MA). Relative mRNA levels were evaluated by quantitative real-time PCR using exon spanning primers, Taqman gene technology and StepOnePlus Real-Time PCR (Applied Biosystems, Foster City, CA) (see **Supplementary Table 1** for primers). Relative quantification was performed with the cycle threshold method (ΔΔCT) normalizing to 18S.

### Isolation of Primary Hepatocytes

Primary hepatocytes were isolated as previously described (36, 37). Briefly, the portal vein was cannulated with a 23 gauge needle and perfused with 70 ml of HBSS with 0.5mM EDTA and 25 mM HEPES. The liver was then perfused 70 ml of a collagenase solution composed of DMEM low glucose media with 15 mM HEPES, 1% penicillin/streptomycin and 100 U/ml of collagenase type IV (Sigma, St Louis, MO). The liver was then removed and plated in a petri dish with culture media, the Glisson’s capsule was ruptured and cells were released. The cell suspension was then poured over a 100 um strainer and placed on ice. The suspension was spun for 9 min at 50 g to pellet the hepatocytes.

### Statistical Analysis

For comparison between exposure groups, the null hypothesis was tested by one-way ANOVA with Dunnett’s correction for multiple comparisons when three or more time points were tested, and by two-tailed Mann-Whitney *t*-test when two time points were evaluated. Statistical significance between groups was defined at p< 0.05 and were analyzed using Prism (Graphpad Software, San Diego, CA).

## Results

### Circulating Selenium and Selenoprotein P Content Are Decreased by Endotoxemia

**Figure 1:**
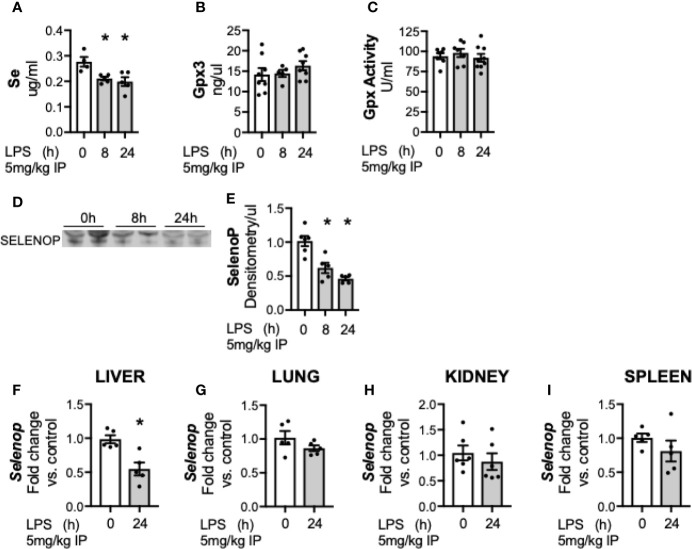
Circulating Se and selenoprotein P (SELENOP) decrease after endotoxemia. Adult male mice were exposed to sublethal LPS (5 mg/kg, IP) and plasma was evaluated at 0, 8, and 24 h. **(A)** Se content by ICP-MS, **(B)** Plasma glutathione peroxidase 3 protein expression by Elisa, **(C)** Plasma glutathione peroxidase activity level by oxidation of NADPH per minute, **(D)** Representative blot of SELENOP in 2 ul plasma **(E)**. Densitometric analysis of SELENOP protein content measured in 2 μl plasma. Whole organ homogenate for hepatic, pulmonary, renal and splenic tissue was evaluated in 0 and 24 h after LPS. Fold changes in mRNA expression are shown normalized to organ control. **(F)** Hepatic *Selenop* mRNA expression, **(G)** Pulmonary *Selenop* mRNA expression, **(H)** Renal *Selenop* mRNA expression, **(I)** Splenic *Selenop* mRNA expression. N = 4–6 for all groups. Data are presented as mean ( ± SEM), *p < 0.05 vs control, by one way ANOVA or two sided t-test.

First, we evaluated the effects of sublethal endotoxemia on circulating Se levels in adult male mice. Plasma levels of Se were significantly lower in endotoxemic mice at 8 and 24 h compared to untreated controls (**Figure 1A**). Circulating Se is primarily incorporated within selenoproteins, and the two main circulating selenoproteins are glutathione peroxidase 3 (Gpx3) and selenoprotein P (SELENOP). To determine how endotoxemia impacted these two major plasma Se-containing compounds, Gpx3 and SELENOP were evaluated. Gpx3 content (**Figure 1B**) and Gpx activity (**Figure 1C**) were unchanged following LPS treatment. In contrast, plasma SELENOP decreased in endotoxemic mice at 8 and 24 h (**Figures 1D, E**) (25). Under homeostatic conditions, the liver is the primary source of circulating SELENOP (38–40). However, *Selenop* mRNA is also expressed by other organs and cell types, and SELENOP expression increases in certain disease states (39, 41). Thus, the transcriptional response to endotoxemia at the 24 h endpoint in the liver, lung, kidney and spleen was subsequently measured. *Selenop* transcription significantly decreased 24 h after LPS treatment in the liver (**Figure 1F**). In contrast, no significant changes were noted in the lung (**Figure 1G**), kidney (**Figure 1H**), or spleen (**Figure 1I**) when compared to respective non-endotoxemic controls.

### Hepatic Transcription and Protein Content of Selenoprotein P Is Decreased by Endotoxemia

**Figure 2:**
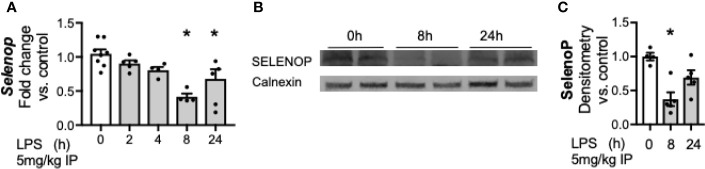
Endotoxemia decreases hepatic transcription and protein content of selenoprotein P. Adult male mice were exposed to sublethal LPS (5 mg/kg, IP) and hepatic whole organ homogenate was evaluated. **(A)** *Selenop* mRNA expression at 0, 2, 4, 8, and 24 h after LPS, **(B)** Representative Western blots of hepatic SELENOP evaluated 0, 8 and 24 h after LPS exposure, **(C)** Densitometric analysis of decrease in SELENOP protein content expression evaluated 0, 8, and 24 h after LPS exposure. Results are normalized to calnexin loading control, and expressed as a ratio to unexposed control mice. N = 4-7 for all groups. Data are presented as mean ( ± SEM), *p < 0.05 vs control, by one way ANOVA.

Having noted a significant decrease in hepatic *Selenop* transcription at 24 h of LPS exposure, we interrogated additional time points to further understand the temporal relationship between LPS exposure and hepatic *Selenop* transcription. *Selenop* transcription was measured at 2, 4, 8, and 24 h after LPS, and decreased by 8 h after exposure (**Figure 2A**). Next, to determine if the decreased hepatic *Selenop* mRNA resulted in similar changes in protein expression, hepatic SELENOP protein levels were measured. We observed to significantly decrease at 8 h of exposure (**Figures 2B, C**).

### Key Hepatic Selenoenzymes Decrease After Endotoxemia

**Figure 3:**
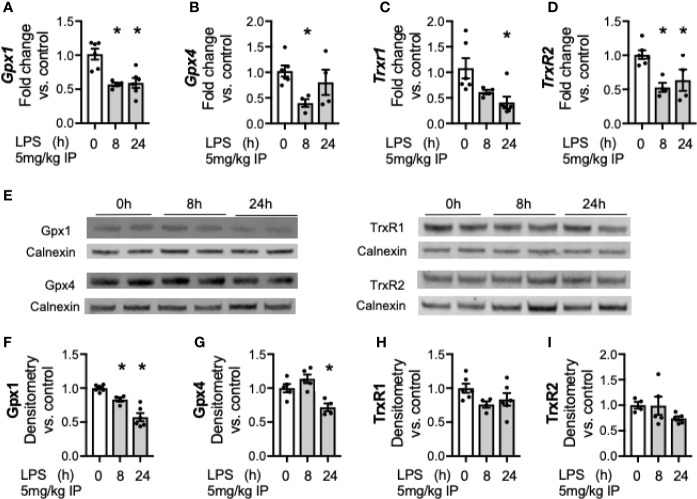
Endotoxemia decreases transcription and protein content of hepatic selenoenzymes. Adult male mice were exposed to sublethal LPS (5 mg/kg, IP) and hepatic whole organ homogenate was evaluated at 0, 8, and 24 h. Fold changes in mRNA expression are shown normalized to unexposed samples. **(A)** *Gpx1* mRNA expression, **(B)** *Gpx4* mRNA expression, **(C)** *TrxR1* mRNA expression, and **(D)** *TrxR2* mRNA expression. **(E)** Representative Western blots of hepatic Gpx1, Gpx4, TrxR1 and TrxR2 after LPS exposure. Densitometric analysis of decrease in **(F)** Gpx1, **(G)** Gpx4, **(H)** TrxR1, and **(I)** TrxR2 protein content expression. Results are normalized to calnexin loading control, and expressed as a ratio to unexposed control mice. N = 4–6 for all groups. Data are presented as mean ( ± SEM), *p < 0.05 vs control, by one way ANOVA or two sided t-test.

After documenting a decrease in hepatic SELENOP after endotoxemia, we tested if LPS exposure decreases other hepatic selenoenzymes. Members of the glutathione peroxidase (Gpx) and thioredoxin reductase (Trxrd) families were assessed. Gpx1 is the most abundant hepatic selenoenzyme, and is considered an essential stress related enzyme during certain inflammatory and oxidative challenges (42). Gpx4 is a phospholipid glutathione peroxidase important in repairing lipid hydroperoxides and regulating ferroptosis (43). The Trxrds are a family of housekeeping redox enzymes essential for thiol disulfide reduction, with Trxrd1 regulating redox state in the cytoplasm and Trxrd2 in the mitochondria (44). LPS decreased hepatic transcription of *Gpx1* (**Figure 3A**), *Gpx4* (**Figure 3B**), *Trxrd1* (**Figure 3C**), and *Trxrd2* (**Figure 3D**) when compared to non-endotoxemic controls. To determine if these transcriptional changes resulted in similar changes in the protein expression, hepatic protein content was measured. LPS decreased the protein content of Gpx1 and Gpx 4 (**Figures 3E–G**). In contrast, Trxrd1 and Trxrd2 expression were unchanged after endotoxemia (**Figures 3E, H–I**).

### Hepatic Proteins Involved in Conversion of Se to the Selenocysteine Are Decreased After Endotoxemia

**Figure 4:**
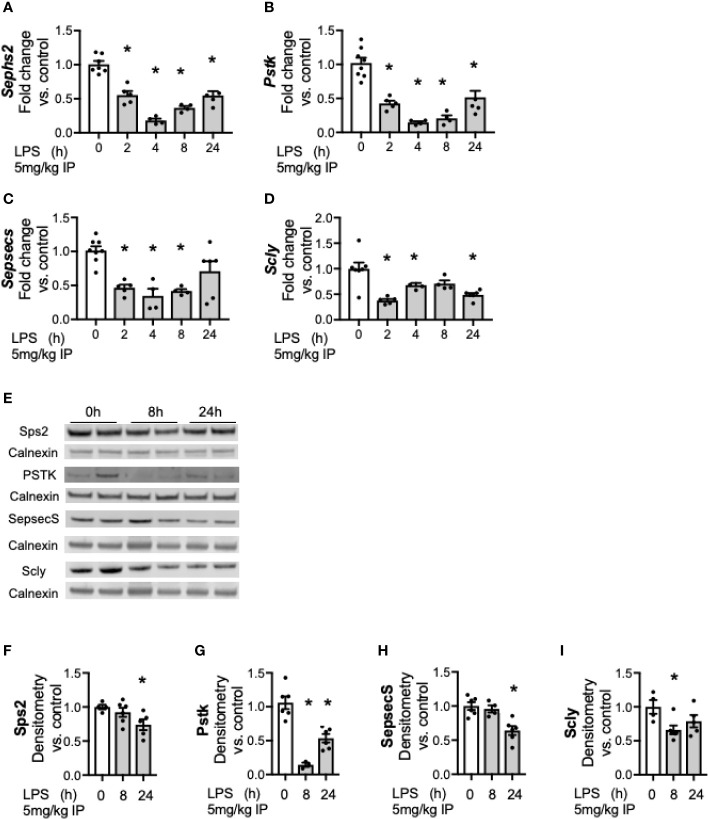
Endotoxemia decreases hepatic factors required for the conversion of Se to bioactive selenocysteine (Sec). Adult male mice were exposed to sublethal LPS (5 mg/kg, IP) and hepatic organ whole homogenate was evaluated at 0, 2, 4, 8, and 24 h. Fold changes in mRNA expression are shown normalized to unexposed samples. **(A)** *Sephs2* mRNA expression, **(B)** *Pstk* mRNA expression, **(C)** *SepsecS* mRNA expression, and **(D)** *Scly* mRNA expression. **(E)** Representative Western blots of hepatic Sps2, Pstk, SepsecS and Scly after LPS exposure. Densitometric analysis of decrease in **(F)** Sps2, **(G)** Pstk, **(H)** SepsecS, and **(I)** Scly protein content expression. Results are normalized to calnexin loading control, and expressed as a ratio to unexposed control mice to calnexin loading control. N = 3–7 for all groups. Data are presented as mean ( ± SEM), *p < 0.05 vs control, by one way ANOVA.

We next tested the expression of proteins and enzymes required to convert Se from diet into its bioactive form. Se must be co-translationally incorporated into the 21^st^ amino acid, selenocysteine (Sec) as it is inserted to nascent selenoproteins and selenoenzymes (34). Generating Sec is a complex multistep process that requires four enzymatic conversions to co-translationally load Se into its specific Sec tRNA prior to incorporation into nascent selenoproteins (34). Prior work has demonstrated that hepatic transcription of these factors decreases during endotoxemia (25). To expand on these earlier published observations and to determine the temporal relationship between LPS exposure and the hepatic transcriptional response, additional time points were evaluated to determine kinetics. Two hours after endotoxin exposure, hepatic transcription of selenophosphate synthetase 2 (*Sephs2*, **Figure 4A**), phosphoseryl tRNA kinase (*Pstk*, **Figure 4B**), selenocysteine synthase (*Sepsecs*, **Figure 4C**) and the selenium recycler protein, selenocysteine lyase (*Scly*, **Figure 4D**) was decreased. This was no longer statistically decreased for *Sepsecs* at 24 h. Hepatic protein content for these factors also decreased in response to endotoxemia (**Figures 4E–I**).

### LPS-Induced Transcriptional Downregulation of Se Processing Factors Occurs in Hepatocytes

**Figure 5:**
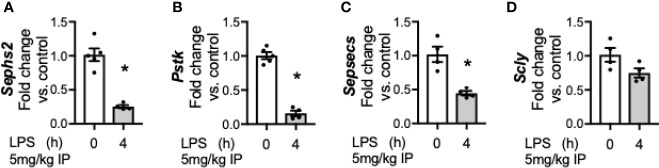
Endotoxemia decreases transcription of factors for selenocysteine processing in the hepatocytes. Adult male mice were exposed to sublethal LPS (5 mg/kg, IP) and hepatocytes were collected and evaluated at 0 and 4 h after LPS. Fold changes in mRNA expression are shown normalized to unexposed samples. **(A)** *Sephs2* mRNA expression, **(B)** *Pstk* mRNA expression, **(C)** *SepsecS* mRNA expression, and **(D)** *Scly* mRNA expression. N = 4-5 for all groups. Data are presented as mean ( ± SEM), *p < 0.05 vs control, by two sided t-test.

After observing a transcriptionally regulated decrease in factors for Se processing in the whole liver after endotoxemia, we evaluated if this process occurred in isolated hepatocytes, as prior work has demonstrated that under physiologic conditions, the hepatocyte is integral in orchestrating Se regulation and transport (39). Hepatocytes were isolated from control mice and mice exposed to endotoxemia at 4 h, as this was a time point all factors were significantly decreased in whole liver homogenate. Similar to the whole liver homogenate results, endotoxemia was associated with a decrease in the mRNA levels of *Sephs2* (**Figure 5A**), *Pstk* (**Figure 5B**), *Sepsecs* (**Figure 5C**) and the selenium recycler protein, *Scly* (**Figure 5D**) within the hepatocyte fraction of the liver.

### Downregulation in Gpx1 and Trxrd1 After Innate Immune Activation With Endotoxemia Does Not Occur in the Lung, Kidney, or Spleen

**Figure 6:**
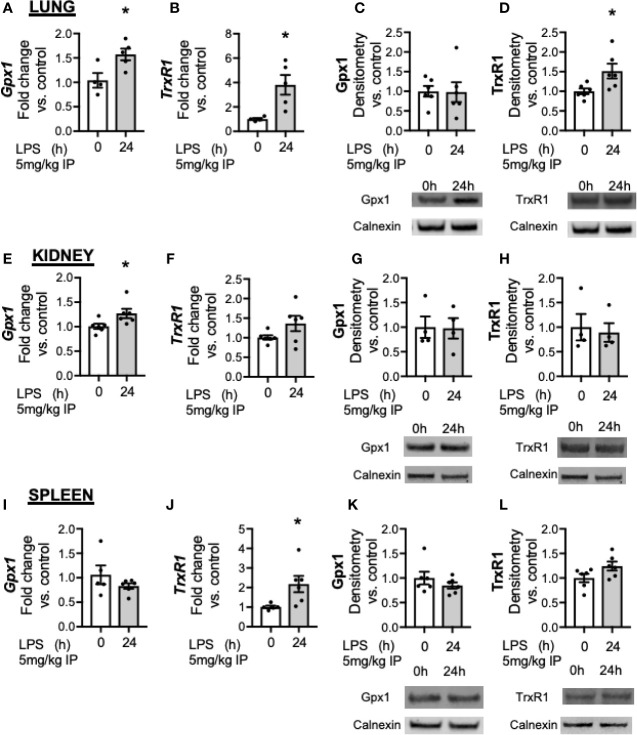
Endotoxemia increases transcription but not protein content of key selenoenzymes in the lung, kidney and spleen. Adult male mice were exposed to sublethal LPS (5 mg/kg, IP) and pulmonary, renal and splenic whole organ homogenate were evaluated 0 and 24 h after LPS. Fold changes in mRNA expression are shown normalized to unexposed samples. **(A)** Pulmonary *Gpx1* mRNA expression, **(B)** Pulmonary *TrxR1* mRNA expression. Representative Western blots and densitometric analysis of expression of **(C)** Pulmonary Gpx1, **(D)** Pulmonary TrxR1. **(E)** Renal *Gpx1* mRNA expression, **(F)** Renal *TrxR1* mRNA expression, Representative Western blots and densitometric analysis of expression of **(G)** Renal Gpx1, **(H)** Renal TrxR1. **(I)** Splenic *Gpx1* mRNA expression, **(J)** Splenic *TrxR1* mRNA expression. Representative Western blots and densitometric analysis of expression of **(K)** Splenic Gpx1, **(L)** Splenic TrxR1. All densitometric analysis has results are normalized to calnexin loading control, and expressed as a ratio to unexposed control mice. N = 4–5 for all groups. Data are presented as mean ( ± SEM), *p < 0.05 vs control, by two sided t-test.

Accumulating literature demonstrates that Se and selenoenzyme status in the lung, kidney and spleen can modulate the response to injury (31–33). While the liver demonstrates the highest abundance of Se, several selenoenzymes are ubiquitously expressed, including Gpx1 and Trxrd1. The impact of innate immune activation with LPS on selenoenzyme status on organs has not been evaluated. Thus, we tested if endotoxemia was associated with decreased Gpx1 or Trxrd1 in the lung, kidney or spleen. In the lung, LPS was associated with increased expression of *Gpx1* (**Figure 6A**) and *Trxrd1* (**Figure 6B**). At a protein level, pulmonary Gpx1 expression was unaltered at 24 h (**Figure 6C**) and Trxrd1 expression was increased (**Figure 6D**). In the kidney, there was an increase in *Gpx1* (**Figure 6E**) but not *TrxR1* transcripts after LPS (**Figure 6F**), and protein expression for both was unaltered (**Figures 6G, H**). In the spleen, LPS did not alter *Gpx1* transcription (**Figure 6I**), and increased *TrxR1* transcription (**Figure 6J**). Proteins levels were not different between groups (**Figures 6K, L**). These results show that in contrast to the liver, there is not a decrease in the transcription or protein expression of glutathione peroxidase 1 in the lung, kidney or spleen.

### Transcriptional Response for Se Processing Factors in the Lung, Kidney, and Spleen Is Dissimilar to the Hepatic Response

**Figure 7:**
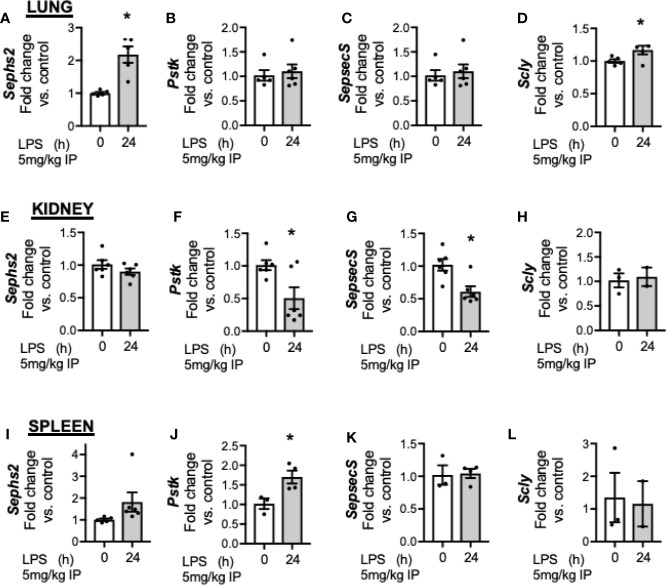
Endotoxemia decreases hepatic transcription and protein content of selenoprotein P. Adult male mice were exposed to sublethal LPS (5 mg/kg, IP) and pulmonary, renal, and splenic whole organ homogenate were evaluated 24 h after LPS. Fold changes in mRNA expression are shown normalized to unexposed samples. **(A)** Pulmonary *Sephs2* mRNA expression, **(B)** Pulmonary *Pstk* mRNA expression, **(C)** Pulmonary *SepsecS* mRNA expression, **(D)** Pulmonary *Scly* mRNA expression, **(E)** Renal *Sephs2* mRNA expression, **(F)** Renal *Pstk* mRNA expression, **(G)** Renal *SepsecS* mRNA expression, **(H)** Renal *Scly* mRNA expression, **(I)** Splenic *Sephs2* mRNA expression, **(J)** Splenic *Pstk* mRNA expression, **(K)** Splenic *SepsecS* mRNA expression, **(L)** Splenic *Scly* mRNA expression (N = 2–5 for all groups. Data are presented as mean ( ± SEM), *p < 0.05 vs control by two sided t-test.

Finally, we evaluated the impact of endotoxemia on factors required for Se processing in the lung, kidney and spleen. In the lung, endotoxemia was associated with increased transcription of *Sephs2* (**Figure 7A**), no change in *Pstk* (**Figure 7B**) or *Sepsecs* (**Figure 7C**) and increased transcription of *Scly* (**Figure 7D**). In the kidney, there was no change in the transcription of *Sephs2* (**Figure 7E**), decreased transcription for *Pstk* (**Figure 7F**) and *Sepsecs* (**Figure 7G**) and no change for *Scly* (**Figure 7H**). Finally, in the spleen, endotoxemia was associated with no change in the transcription of *Sephs2* (**Figure 7I**), an increase in *Pstk* (**Figure 7J**), and no change in *Sepsecs* (**Figure 7K**) or *Scly*, (**Figure 7L**). These results demonstrate that that the transcriptional for factors involved in Se processing is regulated differently in the liver after endotoxemia compared to the lung, kidney and spleen.

## Discussion

Circulating Se levels decrease during clinical sepsis and preclinical models of inflammation, indicating a conserved and shared negative regulatory process may exist for this micronutrient during innate immune challenge (21, 24, 45). Se exerts its biologic effects after a complex multistep co-translational process to incorporate it into selenoproteins. Therefore, to understand Se distribution and processing after innate immune activation with endotoxemia, we tested the expression of key selenoenzymes in the circulation, liver, lung, kidney and spleen. First, we demonstrate the decrease in circulating Se corresponds with a decrease in plasma SELENOP, with no change in Gpx3. Second, we show that endotoxemia decreases the mRNA and protein expression of several hepatic selenoenzymes, but does not decrease these selenoenzymes within the lung, kidney or spleen. Finally, we demonstrate endotoxemia transcriptionally downregulates the hepatic machinery that converts Se to bioactive Sec, with decreases in the mRNA and protein content of multiple cis-regulated enzymes and the Se recycler enzyme. Our temporal assessment shows this acute signaling progress occurs early, with decreased transcription 4 h after endotoxemia. We demonstrate this process occurs in the hepatocytes of mice exposed to endotoxemia. Together, these data support the hypothesis that Se dyshomeostasis occurs in the liver after innate immune activation.

These results establish that LPS induced innate immune activation induces acute and organ specific changes in selenoenzymes and Se processing, with a pattern of downregulation in the liver. The decrease in these hepatic proteins appear to be transcriptionally regulated and to occur early after exposure. Our findings validate the importance of the liver on declining plasma Se status after innate immune activation, corroborating and strengthening those from Renko et al, who first identified that endotoxemia decreases hepatic transcription for *Selenop* and factors involved in Se processing (25). Our study builds on their observations, by defining a time course that shows early transcriptional downregulation with subsequent decreases at the protein level. Additionally, we identify the hepatocyte as a specific cell where this downregulation occurs. After immune challenge, hepatic expression increases for multiple genes and proteins, including cytokines and adhesion markers to recruit inflammatory cells, coagulation factors, as well as proteins involved in the homeostasis of iron and copper, a process collectively referred to as the acute phase response (46–48). Simultaneously, there is a downregulation in other proteins, and our study implicates SELENOP and hepatic Se processing machinery as negatively regulated acute phase proteins in the liver after innate immune activation. Microarray studies revealed that over 200 genes are downregulated after endotoxemia in rodents, thus this downregulation after LPS not exclusive to Se containing enzymes (49). It is currently unclear if the decrease in selenoproteins and factors for Se processing represents an adaptive or advantageous response for the host, or if this process reflects a reprioritization for liver in order to enhance hepatic energy use for the positively regulated acute phase proteins.

SELENOP is a Se enriched protein with 10 Se moieties, and we considered the possibility that hepatic Se is locally redirected for the induction of stress-related hepatic selenoenzymes after endotoxemia. Others have reported endotoxin induces hepatic selenoprotein S, a selenoprotein important in endoplasmic reticulum quality control (26). We do not observe a hepatic induction of several selenoenzymes including Gpx1, Gpx4, Trxrd1, and Trxrd2. These data argue against local redistribution of hepatic Se for these selenoenzyme defense. In contrast, endotoxemia decreases hepatic Gpx transcription and protein content. The dynamic changes to hepatic selenoenzymes after endotoxemia are likely to have clinical implications. Prior work implicates low Se and selenoenzymes in exacerbating injury after hyperoxia, drug toxicity, and viral and bacterial exposures (11–15, 50, 51). We speculate that a persistent deficiency in hepatic selenoenzymes could become maladaptive or predispose to secondary insults.

In contrast to the hepatic downregulation, we found that transcription increased for several of the selenoenzymes in the other organs, with increased protein content for Trxrd1 in the lung. We speculate that this transcriptional increase may support homeostasis of selenoenzyme defenses within the lung, kidney and spleen after innate immune activation. Our results are not surprising, as organ specific, differential expression of selenoenzymes in response to various stimuli. Influenza increases Gpx activity in the lung but decreases its activity in the liver (14). Se deficiency by diet and genetic deletion of the Se transporter protein SELENOP or selenocysteine tRNA (Trsp) alters selenoprotein expression with a complex organ and protein hierarchy. The liver is the first organ to become Se deficient in these models, with a downregulation in stress related selenoenzymes (52, 53). While the mechanisms explaining the organ specific regulation of selenoenzymes during endotoxemia are currently unclear, they highlight the complexity of Se biology.

We speculate that our observation that endotoxemia downregulates factors for Se processing may have implications for restoring Se homeostasis during clinical states that activate TLR4 signaling. Knock-down studies of two of the factors required for Se processing that decrease after endotoxemia, selenophosphate synthetase 2 and phosphoseryl tRNA kinase, are reported to limit production of selenoproteins *in vitro* (25, 54). It is possible that the low hepatic abundance of these factors may have functional implications for Se replacement strategies during inflammation, and could contribute to the failure of Se replacement trials in critically ill adults, however more studies are needed to establish this possibility. TLR4 signaling can be activated in sepsis, but also in sterile inflammation, trauma, and autoimmune diseases (55). Thus, impaired Se processing may occur in a diverse range of clinical scenarios. We observed the transcriptional downregulation for *Sepsecs* was no longer present 24 h after endotoxemia. Additionally, the degree of downregulation did not appear as prominent for *Pstk* or *Sephs2* at 24 h. This may reflect a recovery period occuring in the transcription for Sec processing factors. Future work will need to determine when homeostasis is regained for the circulating and hepatic selenoproteins, as well as hepatic factors for Se metabolism after inflammatory insult. The transcriptional regulation of the factors for Se processing are relatively unclear in mammalian models and this will be an important area of inquiry for our subsequent projects.

A limitation of our study is that we did not perform an exhaustive evaluation of all selenoproteins or organs, and it remains possible that Se is diverted for use in unexamined hepatic selenoproteins or organs not evaluated in our study. Additionally, the mRNA, protein content and activity level of selenoenzymes do not always correlate, and glutathione peroxidase and thioredoxin reductase activity are not evaluated in this study to clarify if endotoxemia induces functional changes. Sex differences are increasingly recognized as important in disease and these experiments utilized male mice only. However, it has been that reported endotoxemia downregulated the transcription of *Pstk*, *Sephs2*, and *Selenop* in both male and female mice, and we speculate the downstream impact on translation occurs in both sexes (25). Finally, we targeted our cell specific inquiry on hepatocytes, and did not examine if the transcriptional responses in Kupffer cells, stellate cells or liver sinusoidal endothelial cells. It is possible the transcriptional downregulation of these factors does occur in other cells types within the liver. Nonetheless, SELENOP hepatocyte-specific knock-out studies implicate the hepatocyte as responsible for the majority of circulating SELENOP. Thus, it is unlikely that any downregulation that occurs in other cell types would confer the same functional significance (39, 40, 56).

## Conclusions

We conclude that innate immune activation with LPS induces transcriptionally regulated, hepatic specific decreases in selenoenzymes and factors required for Se processing. This is temporally associated with decreased circulating Se, mimicking what is observed in clinical sepsis. In contrast to the active downregulation of selenoproteins in the liver, selenoprotein expression was not observed to decrease in the lung, kidney or spleen. Our results have broad clinical implications and highlights the need to consider clinical scenarios in which innate immune activation may decrease the effectiveness of hepatic selenoenzyme defense or Se processing.

## Data Availability Statement

The raw data supporting the conclusions of this article will be made available by the authors, without undue reservation.

## Ethics Statement

The animal study was reviewed and approved by University of Colorado Institute of Animal Care and Use Committee.

## Author Contributions 

LGS, CW, and EN-G conceived and designed the experiments. LGS, LS, and KS contributed to the experiments. LS, CW, EN-G, CD, and TT analyzed the data. LGS wrote the paper. CW, EN-G, TT, CD, and NK revised the manuscript. All authors contributed to the article and approved the submitted version.

## Funding

This study was supported by National Institute of Health/National Heart, Lung and Blood Institute (NIH/NHLBI) R35 HL139726 to EN-G, and NIH/NHLBI HL132941 to CW.

## Conflict of Interest

The authors declare that the research was conducted in the absence of any commercial or financial relationships that could be construed as a potential conflict of interest.
