# Supplementary material for: Hepatic-Specific Decrease in the Expression of Selenoenzymes and Factors Essential for Selenium Processing After Endotoxemia
Source: Front Immunol. 2020 Nov 5;11:595282. doi: 10.3389/fimmu.2020.595282 (PMC7674557; doi:10.3389/fimmu.2020.595282)
Supplement: Supplementary file 1 [file DataSheet_1.docx]

**Supplemental Figure 1: Taqman Primers**

| Gpx1 | Mm00656767_m1 |
| --- | --- |
| Gpx3 | Mm00492427_m1 |
| Gpx4 | Mm00515041_m1 |
| Pstk | Mm00617112_m1 |
| Scly | Mm00489563_m1 |
| Sepp | Mm00486048_m1 |
| SepsecS | Mm00552788_m1 |
| Sephs2 | Mm00545980_s1 |
| TrxR1 | Mm00443675_m1 |
| TrxR2 | Mm00496766_m1 |
